# Microsurgical Management of a Primary Neuroendocrine Tumor of the Filum Terminale: A Surgical Technique

**DOI:** 10.7759/cureus.10080

**Published:** 2020-08-27

**Authors:** Jaime L Martinez Santos, Jeffrey E Wessell, Stephen P Kalhorn

**Affiliations:** 1 Neurosurgery, Medical University of South Carolina, Charleston, USA

**Keywords:** cauda equina, filum terminale, intraoperative ultrasound, neuroendocrine tumor, neuromonitoring, paraganglioma

## Abstract

Neuroendocrine tumors (NETs) are benign tumors of the autonomic nervous system that rarely occur in the spinal canal. The gold standard treatment is gross total resection while preserving the patient’s neurologic functioning as complete surgical resection is curative. The surgical management of NETs could pose a challenge given their friable consistency, hypervascular nature, and proclivity to adhere to the cauda equina nerve roots.

We present a case of a 62-year-old female with an incidental primary NET arising from the filum terminale internum, review the literature, and describe the surgical technique including the benefits of using an intraoperative ultrasound and some of the pitfalls of relying “blindly” on neuromonitoring. Early identification and disconnection of the tumor’s vascular pedicle, which usually runs through the cranial filum, devascularizes the tumor, prevents systemic complications from catecholamine release, and facilitates circumferential dissection off the en passage cauda equina nerve roots. Our patient remains neurologically intact and asymptomatic two years postoperatively and neuroimaging confirmed complete resection.

## Introduction

Neuroendocrine tumors (NETs) or paragangliomas are rare tumors of neural crest origin that occur predominantly in the head and neck, mediastinum, adrenal gland and para-aortic ganglia, and very exceptionally in the spinal canal. Lumbar NETs have been reported and usually arise from preganglionic autonomic cells within the cauda equina nerve roots, accounting for <4% of all tumors in this region [1,2]. A very small subset of these NETs arises from the filum terminale internum (FTI), and gross total resection (GTR) achieves the cure. NETs are hypervascular and friable in consistency, which pose a surgical challenge. We report a case of a NET of the FTI managed surgically with complete resection and discuss the surgical technique.

## Technical report

**Figure 1:**
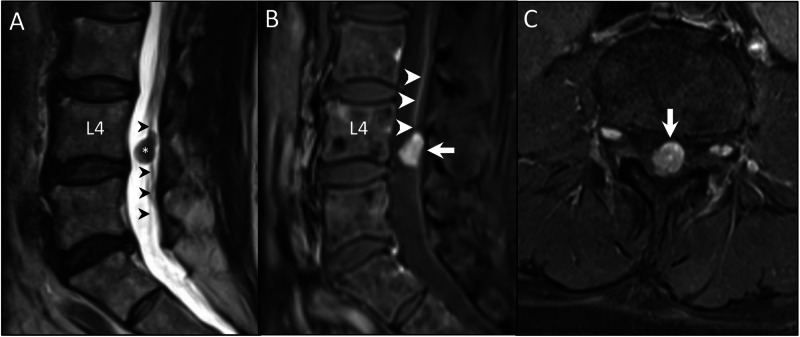
Preoperative MRI showing the tumor (A) T2 MRI revealing an isointense intradural-extramedullary lesion (asterisk) arising from and wrapped around the filum terminale internum (black arrowheads). (B and C) Sagittal and axial, respectively, T1 post-contrast MRI showing a well-demarcated and avidly enhancing (hypervascular) lesion (arrow) at the L4 vertebral level. Note the prominent vascular pedicle going along the cranial filum (white arrowheads).

We present a case of a 62-year-old female with a past medical history of treated hepatitis C and cirrhosis. An abdominal MRI obtained in the workup of hepatic duct dilatation revealed an incidental lumbar spine mass. A dedicated lumbar spine MRI (Figure 1) showed a vividly enhancing lumbar intradural tumor (Figures 1B, 1C) at the level of the L4 vertebral body, arising from and wrapping around the FTI (Figure 1A). She had no back pain or neurological complaints and was neurologically intact on exam. Our initial differential diagnosis included nerve sheath tumor, meningioma, and ependymoma. The patient ultimately decided to have this lesion removed (Video 1) and we performed an L4 laminectomy and GTR of the lesion aided with intraoperative ultrasound (IOUS) and intraoperative neuromonitoring (IONM).

**Video 1:** Microsurgical resection of a neuroendocrine tumor of the filum terminale internum

**Figure 2:**
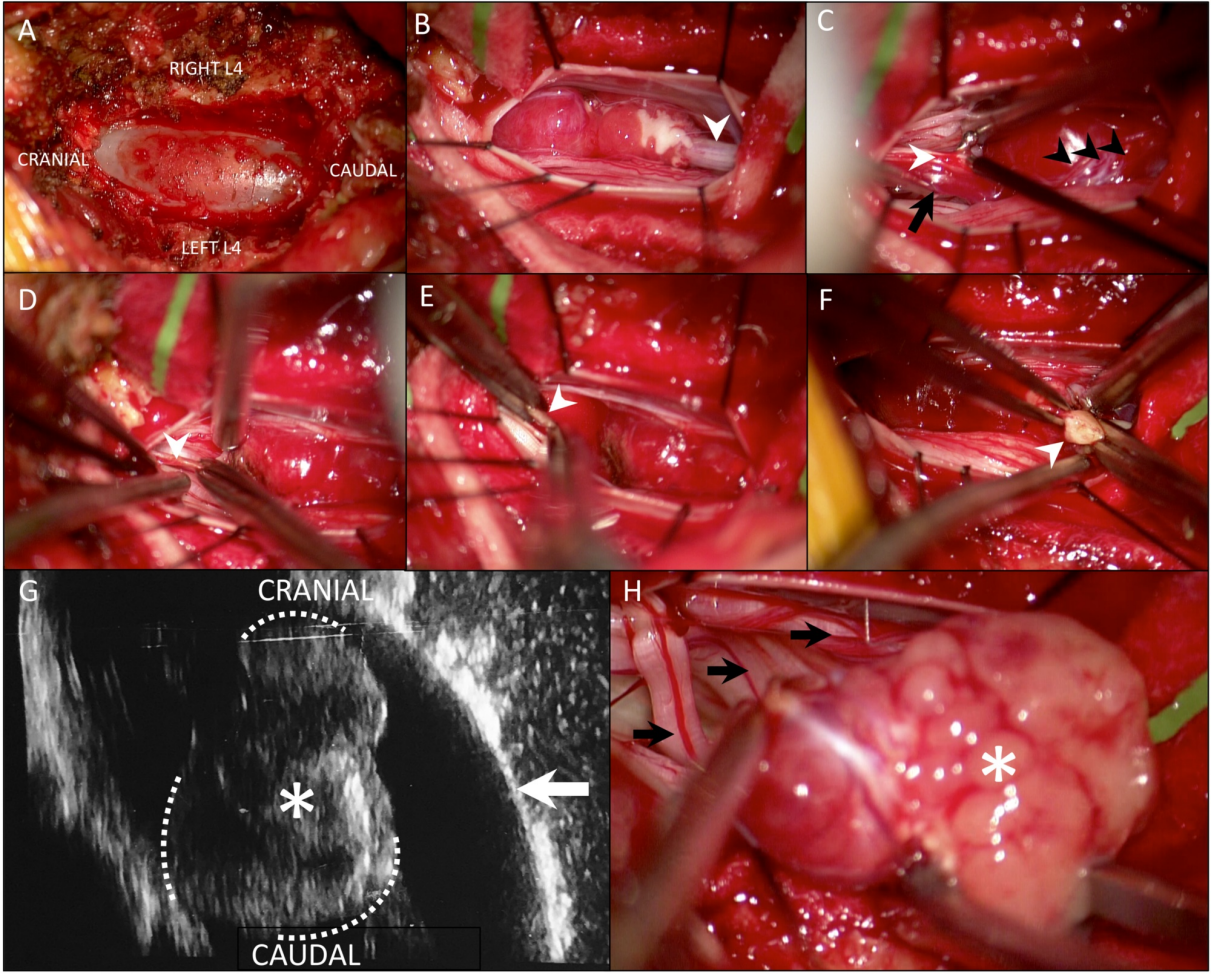
Intraoperative microphotographs and ultrasound image (A) The L4 laminotomy and exposed dura overlying the tumor. (B) The hypervascular tumor arising from the filum (white arrowhead). (C) The cranial filum (white arrowhead) was dissected off the surrounding cauda equina nerve roots, gently pulled up, and stimulated prior to sectioning it (D). Note the prominent vascular pedicle (C, black arrow) going along the filum and the branching network supplying this tumor (C, black arrowheads). (E) A small piece of cranial filum (white arrowhead) was cut and sent for frozen histopathologic analysis and it showed no tumor cells. (F) The caudal filum (white arrowhead) was bipolar electrocoagulated and cut. An intraoperative ultrasound in a longitudinal plane (G) was utilized prior to opening the dura (arrow) and confirmed proper exposure of this tumor (asterisk) cranially and caudally. The ultrasound allowed real-time intraoperative visualization of the tumor margins (dashed lines). (H) Note the color change in the now devascularized tumor (asterisk), which was gently dissected off the en passage cauda equina nerve roots (black arrows) and removed en bloc.

The intraoperative ultrasound (Figure 2G) aided at clearly demarcating this tumor in real time prior to opening the dura (Figure 2A). The dura and arachnoid cranial to the tumor were open and cerebrospinal fluid (CSF) was drained. The tumor’s macroscopic appearance was unlike any other tumor in this region and hypervascular (Figure 2B). It was clearly sprouting from the filum terminale internum (Figures 2B, 2C) and had several en passage cauda equina roots wrapped on its capsule, including the left L5 nerve root.

We identified the major blood supply to the tumor coming along the cranial filum terminale (Figure 2C) and this was coagulated and divided early to devascularize this tumor. Prior to dividing the filum, we stimulated it cranially (Figure 2C) and had electromyographic (EMG) response from the left L5 nerve root at low voltage that we surmised to be spreading of current. The filum was bipolar electrocoagulated and cut (Figure 2D) roughly 5 mm cranial to the tumor. A piece of the attached filum was gently grasped, cut, and sent for frozen histopathology (Figure 2E) to verify that it had no remaining tumor. Once we had confirmation, the filum was allowed to retract cranially. The caudal filum was coagulated and divided (Figure 2F) and we then worked circumferentially, disconnecting small arterial feeders, and gently dissected the tumor off the nerve roots, including the left L5 nerve root that was more closely attached to the anterior surface of the tumor. Finally, the tumor was removed en bloc (Figure 2H). The dura and thoracolumbar fascia were closed watertight. We left a subfascial gravity drain and the patient was kept flat for 36 hours until the drain’s output was minimal.

**Figure 3:**
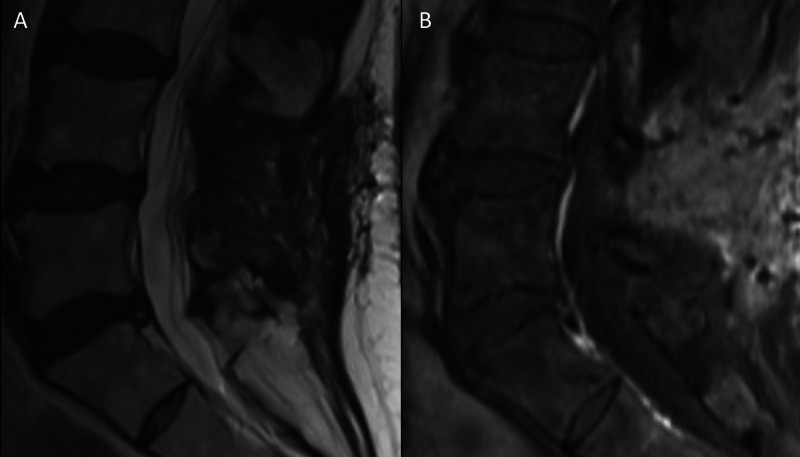
Postoperative MRI showing total resection (A and B) T2 MRI and T1 post-contrast MRI, respectively, showing complete resection and no complication

The patient recovered well and was discharged home on postoperative day 2. Further neuroendocrine workup included chromogranin A testing which was within normal limits. An octreotide scan and MRI of the neuro-axis showed no lesions. A follow-up MRI confirmed total resection (Figure 3). The patient remains asymptomatic at a two-year follow-up.

## Discussion

Neuroendocrine tumors, also known as paragangliomas, are very rare tumors of neural crest origin that arise from autonomic extra-adrenal paraganglia [3]. The term “paraganglia” was first coined by Lerman et al. in 1972 [4], and refers to being in close proximity to the sympathetic ganglia. Paraganglionic cells help regulate fetal blood pressure by secreting catecholamines prior to regressing in childhood [5]. Since these cells are so widespread, NETs can occur nearly anywhere in the body, but the most common locations include the head and neck (glomus tumors), mediastinum, intestines, genitourinary system, para-aortic body (of Zuckerkandl), and very rarely the nervous system and spinal canal.

NETs of the lumbar spine are exceedingly rare, accounting for less than 4% of all tumors in this region [1,2], and typically arise from the cauda equina nerve roots. The proposed cells of origin are autonomic neurons from the intermediolateral cell column (lateral horn) of the spinal cord that migrate along preganglionic nerve root fibers [6,7]. The involvement of the filum would require that local ependymal cells differentiate into neuroendocrine cells, which would be very unusual [8]. Honeyman et al. recently reviewed all cases of lumbar paragangliomas in the literature and reported a total of 296 cases [9]. These tumors are slow growing and become symptomatic by local compression. The most common presenting symptom is chronic back pain (80%-90%) that could be accompanied by radiculopathy (53%), sensory disturbance (22%), motor deficit (25%), or sphincter disturbance (11%-14%) [1,9]. Systemic symptoms form catecholamine release rarely occur with these intradural tumors likely because the small amount of norepinephrine, dopamine, and epinephrine secreted doesn’t reach the threshold concentration in the bloodstream [1,10]. Acute neurological deficits, such as paraplegia and cauda equina syndrome, are rare and usually represent tumor hemorrhage [11], which could be explained by the hypervascular nature of these tumors. In exceptional cases with larger tumors, as it would theoretically occur with any other tumor type in this region, a pressure gradient generated from cerebrospinal fluid drainage (i.e., lumbar puncture, etc.) caudal to the lesion could cause a spinal block and acute neurological deficits [12].

NETs cannot be accurately and reliably differentiated based on neuroimaging alone as there is no pathognomonic finding. There are, however, a number of useful MRI findings attributed to the hypervascular nature of these tumors, venous congestion, and small foci of hemorrhage that aid in the differential diagnosis. Important T2 MRI findings include the classic “salt and pepper” appearance, serpiginous flow voids between the conus medullaris and the lesion, and a peritumoral hypointense hemosiderin rim suggestive of prior hemorrhage [1,2,9,13]. Additionally, NETs have avid contrast enhancement and large vascular pedicles on contrasted MRI sequences.

The definitive diagnosis is based on histopathology with the classic “zellballen” pattern, which are small nests of neuroendocrine chromaffin or “chief cells” with eosinophilic staining surrounded by a fibrovascular stroma containing supporting or “sustentacular cells”. Chief cells stain positively for both chromogranin and synaptophysin while the surrounding sustentacular cells stain positively to S100 on immunohistochemistry [7,14].

NETs of the filum and cauda equina are usually benign, well-encapsulated, and slow-growing World Health Organization (WHO) grade I tumors. The gold standard is surgical GTR, which can be achieved with good margins by removing the filum cranially and caudally, like shown in this case. We also advocate sending a small piece of cranial filum for immediate frozen histopathologic analysis to confirm clean margins as this would dictate if more filum has to be removed. Adjuvant radiation can be considered for any residual tumor. There is currently no role for chemotherapy [2]. Approximately 10%-12% of sub-totally resected lumbar spine NETs will recur at some point, and given the tumor’s slow growth rate, this could occur even 20-30 years after surgery [15-17]. Therefore, long-term follow-up is recommended.

We utilized IOUS and IONM to methodically resect this tumor en bloc through a single level L4 laminectomy. The IOUS helped visualize the tumor in real time and confirmed adequate bony exposure prior to opening the dura [18] as the cranial migration of intradural lumbar tumors relative to the preoperative MRI is well described [19]. The color Doppler function can also be used to localize the vascular pedicle. In this case, the vascular pedicle reached the tumor through the cranial filum. Continuous IONM is helpful at alerting for any excessive spinal cord or nerve root traction during tumor dissection or nerve root ischemia. In this case, direct stimulation on the filum was misleading and resulted in left L5 electromyographic responses, which we surmised was spread of current. Ultimately, the filum was distinguished easily based on anatomy and experience. Early devascularization is key for minimizing intraoperative blood loss from these hypervascular and very friable tumors and should begin by disconnecting the main vascular pedicle at the cranial filum, followed by the caudal filum, and finally any radicular branch supplying the tumor. Once the tumor is devascularized, it can be dissected off the en passage cauda equina nerve roots and then removed en boc. Early devascularization or just clamping the tumor’s vascular pedicle would also prevent intraoperative systemic complications from catecholamine release especially in vulnerable patients with cardiac history and limited cardiac reserve. Preoperative endovascular embolization is an option [20], particularly in cases in which the tumor cannot be resected en bloc or a residuum is anticipated. However, intraoperative hemostasis in these cases is straightforward in our experience.

## Conclusions

We present the successful microsurgical management of a NET originating from the FTI. GTR is curative and early devascularization is key for safe and effective resection. In this case, the tumor’s vascular pedicle reached the tumor through the cranial FT.
